# Supplementary material for: Effect of 1α,25(OH)2 Vitamin D3 in Mutant P53 Glioblastoma Cells: Involvement of Neutral Sphingomyelinase1
Source: Cancers (Basel). 2020 Oct 28;12(11):3163. doi: 10.3390/cancers12113163 (PMC7694157; doi:10.3390/cancers12113163)
Supplement: Supplementary file 1 [file cancers-12-03163-s001.pdf]

SupplementaryMaterials:

Effect of 1α,25(OH)$_2$ Vitamin D$_3$ in Mutant P53 Glioblastoma Cells: Involvement of Neutral Sphingomyelinase1

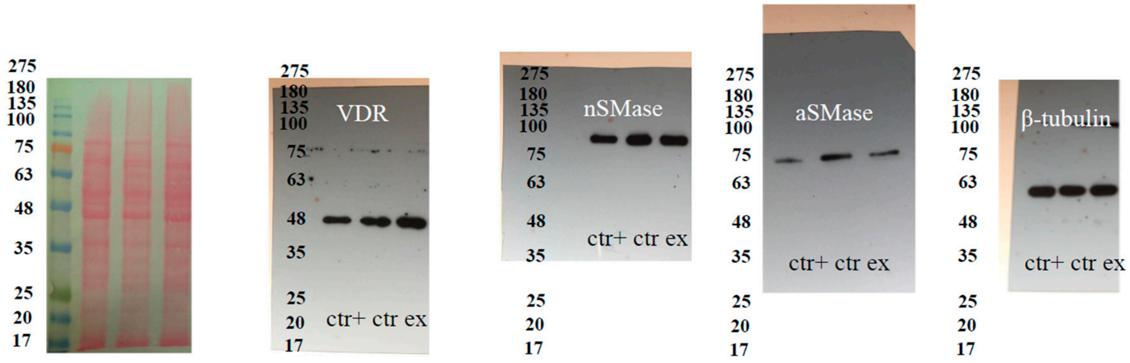

Figure S1. Protein expression in GL15 cells treated with 100nM 1α,25(OH)$_2$ Vitamin D$_3$
Ctr+, positive control (HN9.10 cells); ctr, control sample; ex, experimental sample

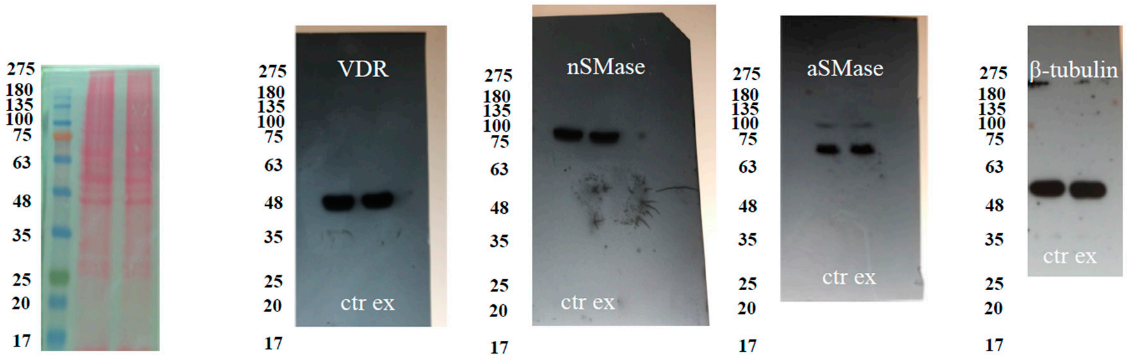

Figure S2. Protein expression in GL15 cells treated with 400nM 1α,25(OH)$_2$ Vitamin D$_3$
ctr, control sample; ex, experimental sample

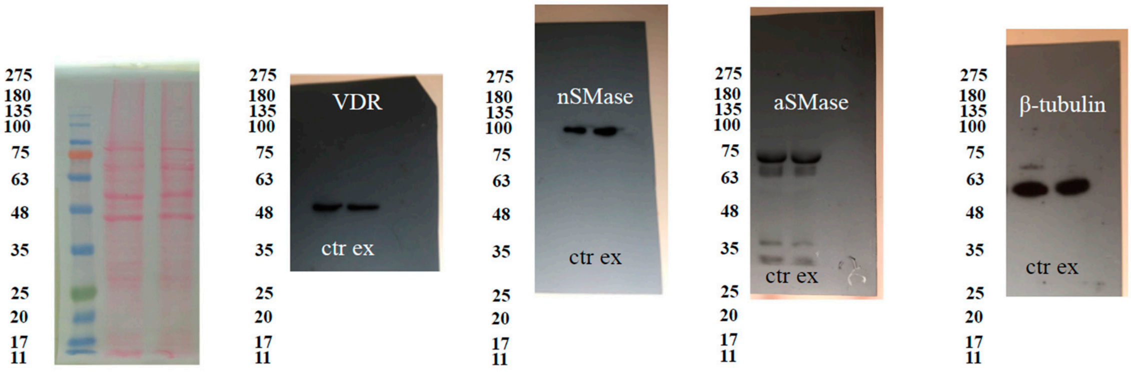

Figure S3. Protein expression in U251 cells treated with 100nM 1α,25(OH)$_2$ Vitamin D$_3$
ctr, control sample; ex, experimental sample

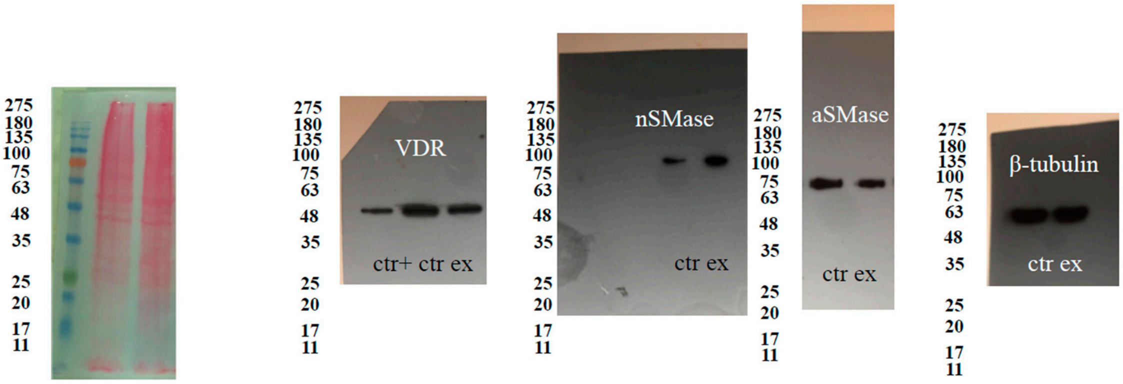

Figure S4. Protein expression in U251 cells treated with 400nM 1α,25(OH)$_2$ Vitamin D$_3$.
Ctr+, positive control (HN9.10 cells); ctr, control sample; ex, experimental sample

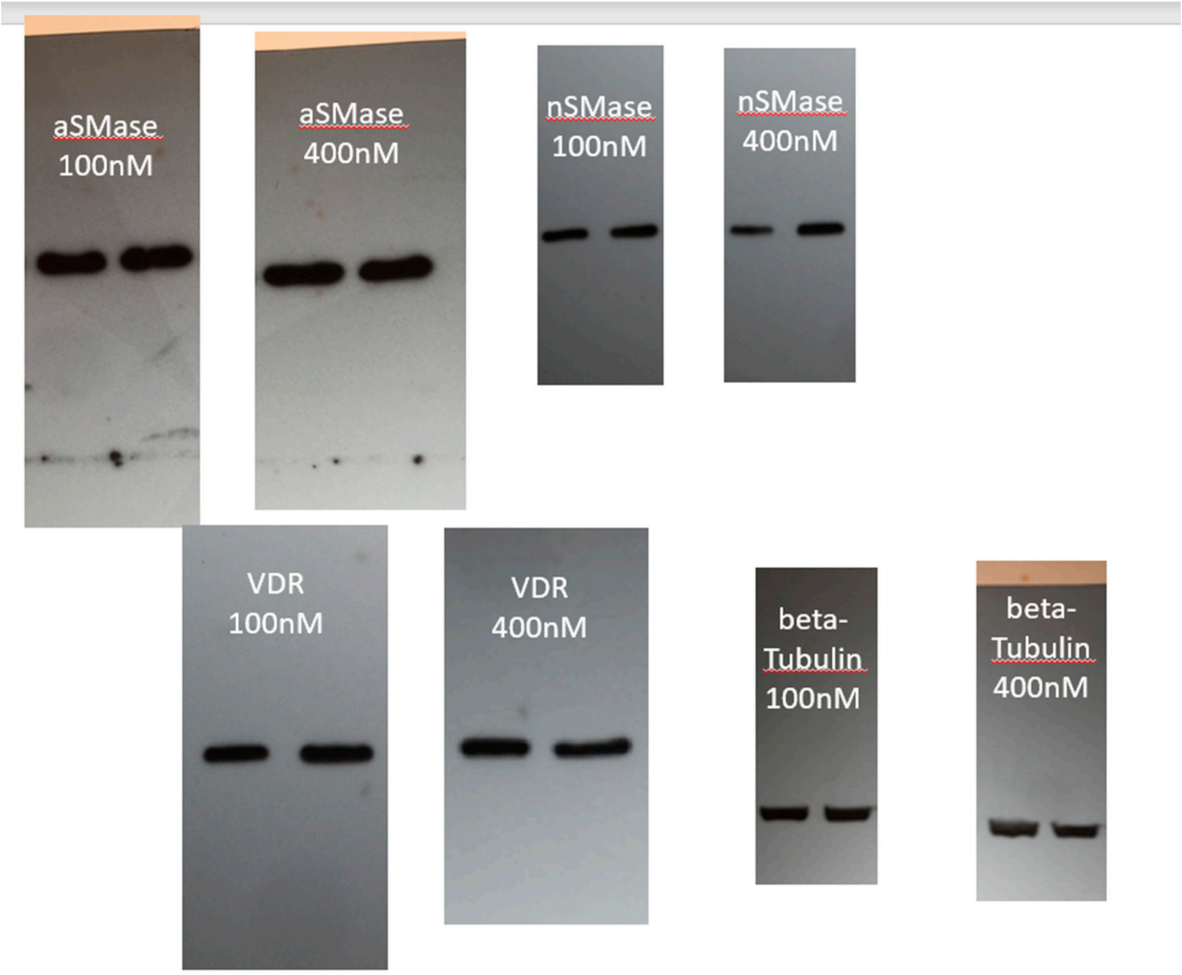

Figure S5. Protein expression in LN18 cells treated with 100 and 400nM 1α,25(OH)$_2$ Vitamin D$_3$.
ctr, control sample; ex, experimental sample
